# Triglyceride-Glucose Index and Intravenous Thrombolysis Outcomes for Acute Ischemic Stroke: A Multicenter Prospective–Cohort Study

**DOI:** 10.3389/fneur.2022.737441

**Published:** 2022-02-14

**Authors:** Sheng-Feng Lin, Han-Hwa Hu, Hai-Lun Chao, Bo-Lin Ho, Chih-Hung Chen, Lung Chan, Huey-Juan Lin, Yu Sun, Yung-Yang Lin, Po-Lin Chen, Shinn-Kuang Lin, Cheng-Yu Wei, Yu-Te Lin, Jiunn-Tay Lee, A-Ching Chao

**Affiliations:** ^1^Department of Public Health, School of Medicine, College of Medicine, Taipei Medical University, Taipei, Taiwan; ^2^School of Public Health, College of Public Health, Taipei Medical University, Taipei, Taiwan; ^3^Department of Emergency Medicine, Taipei Medical University Hospital, Taipei, Taiwan; ^4^Department of Critical Care Medicine, Taipei Medical University Hospital, Taipei, Taiwan; ^5^Beijing Tiantan Hospital, Capital Medical University, Beijing, China; ^6^Advanced Innovation Center for Human Brain Protection, Capital Medical University, Beijing, China; ^7^Department of Neurology, Taipei Medical University-Shaung Ho Hospital, Taipei, Taiwan; ^8^Department of Optometry, Chung Hwa University of Medical Technology, Tainan, Taiwan; ^9^Department of Neurology, Kaohsiung Medical University, Kaohsiung, Taiwan; ^10^Department of Neurology, Kaohsiung Medical University Hospital, Kaohsiung, Taiwan; ^11^Department of Neurology, National Cheng Kung University Hospital, Tainan, Taiwan; ^12^Department of Neurology, National Cheng Kung University, Tainan, Taiwan; ^13^Department of Neurology, Chi Mei Medical Center, Tainan, Taiwan; ^14^Department of Neurology, En Chu Kong Hospital, New Taipei City, Taiwan; ^15^Department of Neurology, Taipei Veterans General Hospital, Taipei, Taiwan; ^16^Department of Neurology, Taichung Veterans General Hospital, Taichung, Taiwan; ^17^Stroke Center and Department of Neurology, Taipei Tzu Chi Hospital, Buddhist Tzu Chi Medical Foundation, New Taipei City, Taiwan; ^18^Department of Neurology, Show Chwan Memorial Hospital, Changhua, Taiwan; ^19^Division of Neurology, Department of Medicine, Kaohsiung Veterans General, Kaohsiung, Taiwan; ^20^Department of Neurology, Tri-Service General Hospital, National Defense Medical Center, Taipei, Taiwan

**Keywords:** acute ischemic stroke, triglyceride, triglyceride-glucose index, intravenous thrombolysis, symptomatic intracranial hemorrhage

## Abstract

**Background:**

The triglyceride-glucose (TyG) index has recently been proposed as a reliable marker of insulin resistance. There is insufficient evidence to verify that the TyG index is correlated with functional outcomes and hemorrhagic transformation and in patients with stroke treated with intravenous thrombolysis (IVT).

**Methods:**

We designed a multicenter cohort study, which enrolled patients with acute ischemic stroke treated with IVT between December 2004 and December 2016. The TyG index was divided into tertiles and calculated on a continuous scale. Unfavorable functional outcomes were defined by the modified Rankin Scale of 3–6 at 90 days and the incident rates of symptomatic intracranial hemorrhage (SICH) within 36 h of IVT onset were surveyed. Stroke severity was defined as mild (4–8), moderate (9–15), or high (≥16) based on the National Institutes of Health Stroke Scale (NIHSS) scores.

**Results:**

Among 914 enrolled patients, the tertiles of the TyG index were 8.48 for T1, 8.48–9.04 for T2, and 9.04 for T3. T3 showed an increased risk of unfavorable functional outcomes at 90 days [odds ratio (OR): 1.76; *P* = 0.0132]. The TyG index was significantly associated with unfavorable functional outcomes at 90 days (OR: 1.32; *P* = 0.0431 per unit increase). No association was found between the TyG index and SICH. These findings were applicable for T3 with stroke of moderate (OR, 2.35; *P* = 0.0465) and high severity (OR: 2.57, *P* = 0.0440) patients with stroke.

**Conclusion:**

This study supports the strong association between the increased TyG index and increased unfavorable functional outcomes at 90 days in patients with acute ischemic stroke treated with IVT. These findings were found to be robust in patients with moderate and high stroke severity.

## Introduction

Early detection of insulin resistance (IR) is crucial in preventing cardiovascular diseases (1, 2). Glucose clamp tests are performed in the two ways: (1) the hyperglycemic clamp technique for quantification of beta-cell sensitivity to glucose and (2) the euglycemic insulin clamp technique for quantification of peripheral cell sensitivity to insulin (3). These techniques are regarded as the gold standard for quantifying IR (4) and rely on constant insulin infusion and measurement of glucose disposal under steady-state conditions (3, 4). The limitations of the glucose clamp technique include labor-intensive and time-consuming procedures, which require experienced physicians to perform human studies. In 1985, a simple equation homeostasis model assessment-IR (HOMA-IR) (5) was developed to assess IR by calculating the product of fasting levels of insulin and glucose (6). Although widely used in the research field, the use of HOMA-IR is greatly limited in clinical practice because of the need for insulin measurement.

Recently, the triglyceride-glucose (TyG) index has been recognized as a reliable surrogate biomarker of IR (7) and has been proposed and validated to be highly correlated with HOMA-IR across all the ages and various ethnic groups (7–10). The TyG index was used for the first time in 2008 by Simental-Mendia et al. (11) to identify IR in an apparently healthy population and it was formally proposed by Guerrero-Romero et al. (12) in 2010. The rationale for the application of the TyG index is that IR is the most common cause of increased triglyceride (TG) and glucose levels in serum tests (13). Some studies showed high correlation between the TyG index and IR by glucose clamp tests (9, 14) and a study (9) showed that the TyG index exhibited higher diagnostic performance than HOMA-IR for IR in some studies. Furthermore, there has been increasing evidence supporting the correlation between the TyG index and acute adverse outcomes in patients with cardiovascular disease. Recent studies have suggested that the TyG index is highly associated with carotid atherosclerosis (15) and the outcomes and prevalence of coronary artery diseases (16, 17). By using HOMA-IR, previous studies have found that IR is associated with neurological worsening and functional status (18–20). In addition, a recent study (21) found that an increased TyG index was associated with a higher risk of stroke recurrence, functional worsening, and mortality. The above studies suggest that the TyG index is a marker for acute adverse effects in cardiovascular and cerebrovascular diseases.

Nonetheless, relevant investigations of the association between the TyG index and intravenous thrombolytic outcomes for stroke are lacking. The Taiwan Thrombolytic Therapy for Acute Ischemic Stroke (TTT-AIS) registry contains a nationwide cohort of Taiwan and longitudinal follow-up data for patients with stroke treated with intravenous thrombolysis (IVT) (22, 23). The TTT-AIS registry comprehensively enrolls patients with acute ischemic stroke with all the levels of severity. The aim of this study was to investigate the relationship between the TyG index and outcomes of functional status and symptomatic intracranial hemorrhage among ethnic Chinese patients with acute ischemic stroke treated with IVT. The novelty and significance of this study are as follows: (1) evaluation of patients with acute ischemic stroke in different categories of the TyG index, (2) nationwide, multicenter cohort study design encompassing the representative population, and (3) evaluation of patients with acute ischemic stroke of all the levels of severity.

## Methods

### Study Design

This study had a prospective cohort design and encompassed a multicenter study of 30 hospitals in Taiwan. Clinical data were collected prospectively and registered in the TTT-AIS registry. Baseline demographic information included age, sex, alcohol use, history of hypertension, diabetes mellitus, coronary artery disease, atrial fibrillation, blood pressure on arrival at the hospital, use of antiplatelet and anticoagulant medications, the National Institutes of Health Stroke Scale (NIHSS) score at baseline, and time from stroke onset to IVT was retrieved by the investigators. For patients with acute ischemic stroke arriving at the hospital within 3 h of stroke onset, IV alteplase was used for the thrombolytic regimen. Serum glucose levels and lipid profiles were obtained from each patient after an overnight fast ≥8 h within 24–48 h of stroke onset. A written informed consent was obtained from all the patients. This study was approved by the Institutional Review Board of the Kaohsiung Medical University Hospital (reference number: KMUH-IRB-20140305).

### Participants

The TyG index was calculated by using the following formula: Ln [TG (mg/dl) × fasting glucose (mg/dl)/2]. According to the previous studies (24, 25), a simple cutoff of the TyG index ≥8.4 is sufficiently reliable to classify Asian individuals with IR. The inclusion criteria for eligible patients were as follows: (1) treatment with IVT for acute ischemic stroke adhering to the National Institute of Neurological Disorders and Stroke (NINDS) criteria (26) and (2) measurement of fasting glucose and lipid profiles in a fasting state during 24–72 h following the administration of IV alteplase. The exclusion criteria for IVT were based on the Safe Implementation of Thrombolysis in Stroke-Monitoring Study (SITS-MOST) study criteria (27). All the enrolled patients with stroke underwent brain CT on arrival to the hospital and another routine brain CT was performed within 24–36 h post-IVT.

### Outcomes Measures

We evaluated the clinical outcomes of (1) unfavorable functional outcome status defined by the modified Rankin Scale (mRS) of 3–6 at 90 days; (2) mortality at 90 days; (3) symptomatic intracranial hemorrhage (SICH); (4) the NINDS standard as per which any intracranial hemorrhage deteriorated to the NIHSS score of ≥ 1 or led to death within 36 h (26); (5) the European Cooperative Acute Stroke Study (ECASS) II standard, as per which any intracranial hemorrhage deteriorated to the NIHSS score of ≥ 4 or led to death (28); and (6) the SITS-MOST standard for a type 2 parenchymal hemorrhage (a local or remote parenchymal intracranial hemorrhage exceeding 30% of the infarct) with clinical deterioration of the NIHSS score of ≥ 4 or death within 36 h (27). Patients who presented with the baseline NIHSS scores of 4–8, 9–15, and ≥ 16 were categorized into the mild, moderate, and high severity groups, respectively.

### Statistical Analysis

To compare the groups with and without IR, the Student's *t*-test was used for continuous variables and the Pearson's chi-squared test was used for categorical variables. We evaluated the relationship between the TyG index and lipid profiles by using two methods: (1) partitioning of the TyG index to the territorial scale and (2) examination of the TyG index on a continuous scale. The multiple logistic regression models were employed to determine the odds ratios (ORs) and their 95% CIs, the study outcomes were used as dependent variables, and the TyG index (either in tertile or in continuous scale) and the unbalanced covariates between the groups with and without IR were independent variables. T1 was used as the reference group for the models analyzed on a tertile scale. As a sensitivity analysis, we performed a stratified analysis according to the stroke severity of each patient. Statistical significance was defined as *p* < 0.05. All the analyses were performed by using SAS software (version 9.4; North Carolina, USA) and Stata software (version 15; Texas, USA).

### Sample Size Estimation

At the time of designing this study, there had been no published study investigating the relationship between the TyG index and clinical outcomes in patients with acute ischemic stroke treated with IVT. The required sample size was estimated in a previous study (29), which explored the relationship between HOMA-IR and clinical outcomes in patients with stroke treated with IVT. In this study (29), HOMA-IR in the upper tertile (OR, 8.54, 95% CI, 1.67–43.55; *P* = 0.01) was associated with unfavorable functional outcome when compared with the lower tertile and HOMA-IR in the middle tertile (OR, 2.96, 95% CI, 0.61–14.40; *P* = 0.178) was not significantly associated with unfavorable functional outcome. Therefore, the required sample size should be 101; when we hypothesized that the parameters of effect size of OR were in a range from 2.96 to 8.54, the probability of exposure (the upper tertile) was 0.33 and significance level and power were 0.05 and 0.95, respectively.

## Results

### Baseline Characteristics

From 1st December, 2004 to 31st December, 2016, a total of 914 patients with acute ischemic stroke who had completed IVT were enrolled and laboratory tests for glucose and lipid profile were performed in a fasting state following admission. Of these, 652 patients had IR (TyG index ≥ 8.4) and 262 patients did not had IR (TyG index < 8.4). In the groups without and with IR (Table 1), the average age was 69.8 ± 13.0 and 68.8 ± 11.9 years (*P* = 0.2941), the proportion of female sex was 36.6 and 36.2% (*P* = 0.8994), the average dose of alteplase was 0.78 ± 0.14 and 0.80 ± 0.14 (*P* = 0.0747), and the mean NIHSS score at onset was 14.2 ± 6.6 and 13.6 ± 7.4 (*P* = 0.2809), respectively; there was no significant difference in the antithrombotic medication usage. The systolic (161.6 ± 29.6 vs. 154.1 ± 29.1 mm Hg, *P* = 0.0011) and diastolic (91.4 ± 19.7 vs. 87.6 ± 18.3 mm Hg, *P* = 0.0078) blood pressures were noted for the group with IR. Medical comorbidities of hypertension and diabetes mellitus were higher in the group with IR than in the group without IR, while the prevalence of atrial fibrillation was higher in the group without IR.

**Table 1:** Demographic characteristics of patients.

**Variable**	**No Insulin Resistance** **(TyG <8.4)** **(*N =* 262)**	**Insulin resistance** **(TyG ≥8.4)** **(*N =* 652)**	***p*-value**
Age (years)	69.8 ± 13.0	68.8 ± 11.9	0.2941
Age groups (years)			0.5287
20–39 years	2.3% (6/262)	1.2% (8/652)	
40–49 years	3.1% (8/262)	3.5% (23/652)	
50–59 years	16.0% (42/262)	17.9% (117/652)	
60–69 years	21.4% (56/262)	25.8% (168/652)	
70–79 years	33.2% (87/262)	31.8% (207/652)	
80–89 years	21.4% (56/262)	17.5% (114/652)	
≥ 90 years	2.7% (7/262)	2.3% (15/652)	
Female sex; *n* (%)	36.6% (96/262)	36.2% (236/652)	0.8994
Alcoholism; *n*/total *N* (%)	25/262 (9.5%)	61/652 (9.4%)	0.9305
Mean NIHSS on arrival	14.2 ± 6.6	13.6 ± 7.4	0.2809
Stroke Severity at baseline			0.1678
Mild (NIHSS of 4–8)	72/262 (27.5%)	178/652 (27.3%)	
Moderate (NIHSS of 9–15)	80/262 (30.5%)	238/652 (36.5%)	
High (NIHSS of ≥ 16)	110/262 (42.0%)	236/652 (36.2%)	
Alteplase dose (mg/kg)	0.78 ± 0.14	0.80 ± 0.14	0.0747
Groups of Alteplase dosage			0.9898
Standard dose (0.9 mg/kg)	67/262 (25.6%)	167/652 (25.6%)	
Low dose (<0.9 mg/kg)	195/262 (74.4%)	485/652 (74.4%)	
**Blood pressure on arrival**			
Systolic BP (mmHg)	154.1 ± 29.1	161.6 ± 29.6	0.0011^*^
Diastolic BP (mmHg)	87.6 ± 18.3	91.4 ± 19.7	0.0078^*^
Time to treatment (min)	131.3 ± 46.7	130.6 ± 46.4	0.8441
Medical history			
Hypertension	168/262 (64.1%)	508/652 (77.9%)	<0.0001^*^
Diabetes mellitus	37/262 (14.1%)	279/652 (42.8%)	<0.0001^*^
Coronary artery disease	35/262 (13.4%)	105/652 (16.1%)	0.2973
Atrial fibrillations	131/208 (63.0%)	268/501 (53.5%)	0.0204^*^
Antithrombotic use			
Aspirin	33/148 (22.3%)	85/365 (23.3%)	0.8092
Clopidogrel	4/148 (2.7%)	18/365 (4.9%)	0.2589
Ticlopidine	1/148 (0.7%)	1/365 (0.3%)	0.4942
Warfarin	8/148 (1.6%)	12/365 (3.3%)	0.3133
**Metabolism markers**			
Fasting glucose	103.5 ± 25.8	153.3 ± 115.2	<0.0001^*^
Lipids (mg/dL)			
Total cholesterol	160.9 ± 39.3	191.7 ± 46.9	<0.0001^*^
LDL-C	95.2 ± 33.4	115.7 ± 42.5	<0.0001^*^
HDL-C	49.6 ± 20.9	48.1 ± 25.4	0.3985
TG	64.7 ± 19.0	147.4 ± 82.9	<0.0001^*^

*BP, blood pressure; HDL-C, high density lipoprotein-cholesterol; LDL-C, low density lipoprotein-cholesterol; NIHSS, National Institutes of Health Stroke Scale; TG, triglyceride; TyG, triglyceride-glucose index*.

*Continuous variables are expressed as the mean ± SD*.

* *Statistically significant at p < 0.0*.

### Functional Outcome Distribution by the TyG Index

**Figure 1:**
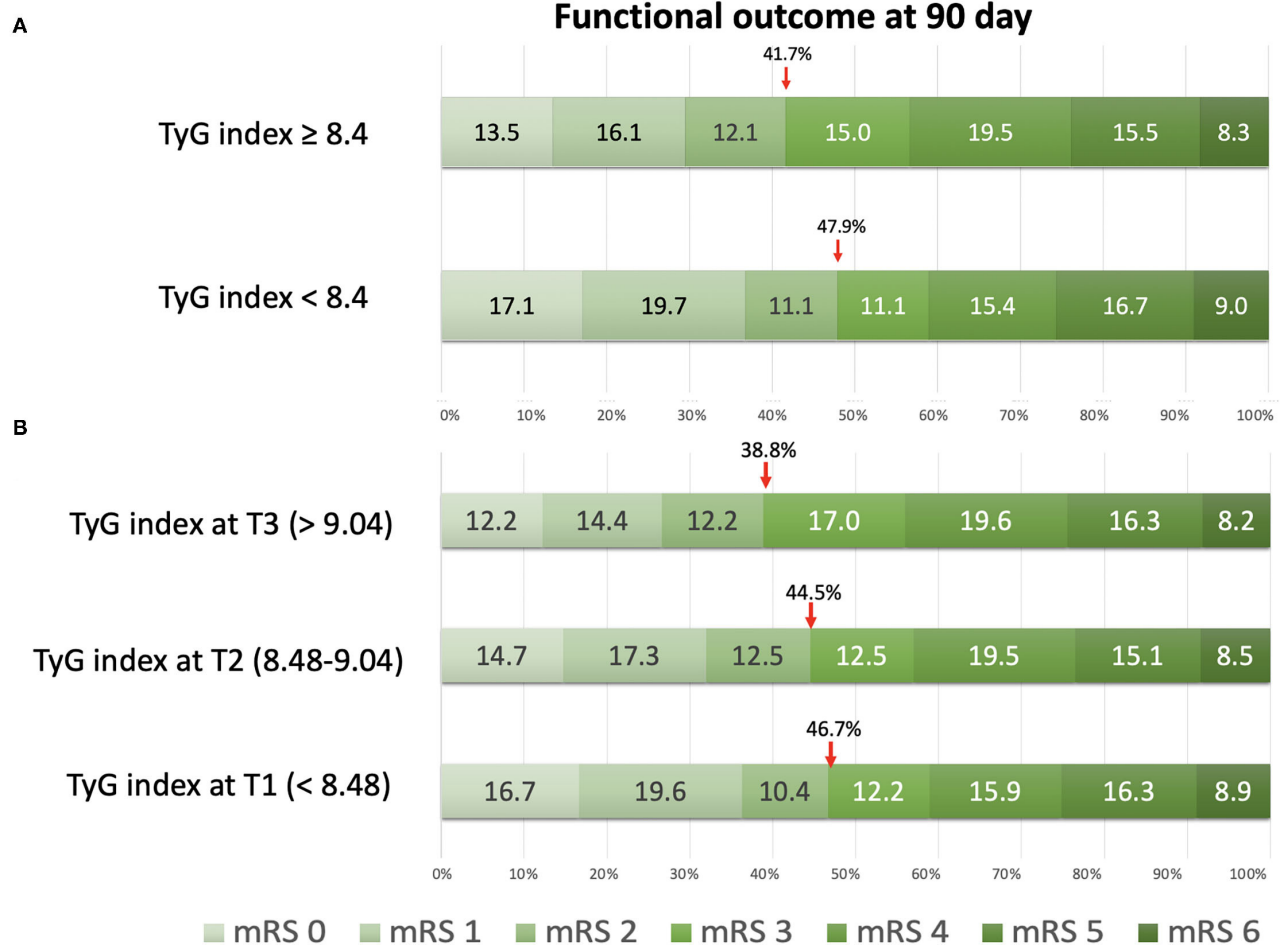
The distribution of functional outcomes at 90 days. **(A)** Classification by insulin resistance status with the triglyceride-glucose (TyG) index at 8.4. **(B)** Classification by tertiles of the TyG index.

The functional outcome distribution defined by the mRS at 90 days is shown in Figure 1. Patients with stroke in the group without IR showed higher proportions of favorable outcome (mRS, 0–2) at 90 days (47.9%) than those patients with stroke in the group with IR (41.7%). The cutoff value of the TyG index on the tertiles was T1 < 8.48, T2 ranging between 8.48 and 9.04, and T3 > 9.04, respectively. On classification by tertiles, the proportions of favorable functional outcomes at 90 days decreased with increasing tertiles (T1, 46.7%; T2, 44.5%; and T3, 38.8%).

### Outcomes by the TyG Index on the Tertile Scale

**Figure 2:**
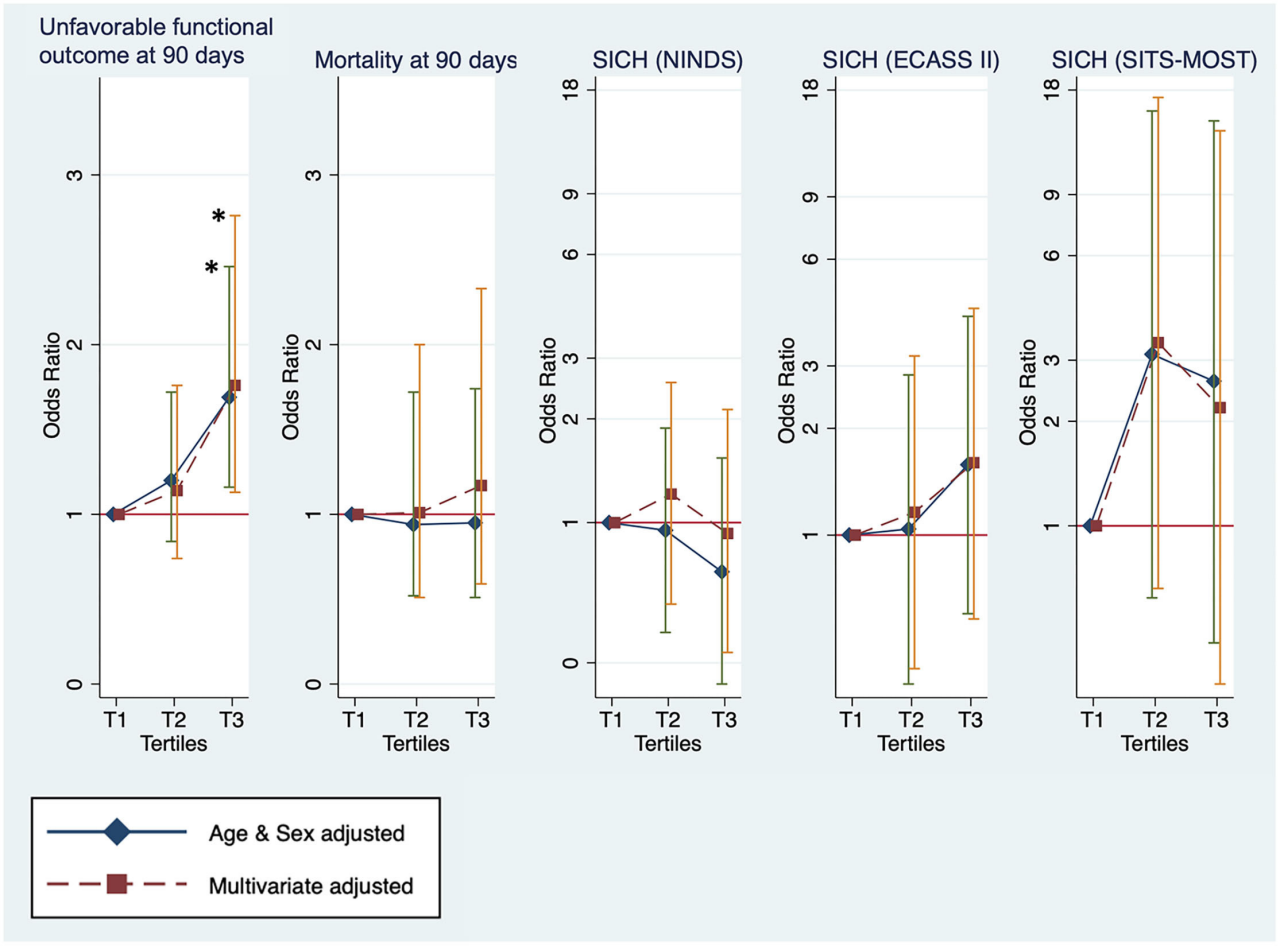
The outcomes measures for unfavorable functional outcome status and mortality at 90 days and symptomatic intracranial hemorrhage (SICH) by tertiles of the TyG index. *Statistical significance (p < 0.05).

Clinical outcomes that were investigated by categorizing patients with stroke into tertiles of the TyG index are shown in Figure 2. For unfavorable functional outcomes at 90 days, T3 had the highest event rate of 73.6%, compared to T1 (63.8%) and T2 (68.0%) (Table 2). After adjustment for age and sex, the logistic regression model showed a significant increase in unfavorable functional outcomes at 90 days for T3 (OR, 1.69; 95% CI, 1.16–2.46; *P* = 0.0059). In the multivariate-adjusted models, T3 showed a significantly increased risk of unfavorable functional outcomes at 90 days (OR, 1.76; 95% CI, 1.13–2.76; *P* = 0.0132). All the tertiles showed similar rates for mortality, ranging from 8.2 to 8.9%. Additionally, there was no significant difference in the rates of SICH according to the NINDS, the ECASS II, and the SITS-MOST criteria.

**Table 2:** Functional outcomes and symptomatic intracranial hemorrhage (SICH) by tertiles of the triglyceride-glucose (TyG) index.

**Models**	**TyG**	***N***	**Events (%)**	**Age, sex- adjusted of TyG index**		**Multivariable- adjusted^†^** **of TyG index**	
**Variable**				**OR (95% CI)**	***p*-value**	**OR (95% CI)**	***p*-value**
Unfavorable functional outcome at 90 days	T1	271	63.8% (173/271)	1.00	–	1.00	–
	T2	272	68.0% (185/272)	1.20 (0.84–1.72)	0.3233	1.14 (0.74–1.76)	0.5455
	T3	269	73.6% (198/269)	1.69 (1.16–2.46)	0.0059^*^	1.76 (1.13–2.76)	0.0132^*^
Mortality at 90 days	T1	271	8.9% (24/271)	1.00	–	1.00	–
	T2	272	8.5% (23/272)	0.94 (0.52–1.72)	0.8491	1.01 (0.51–2.00)	0.9746
	T3	269	8.2% (22/269)	0.95 (0.51–1.74)	0.8542	1.17 (0.59–2.33)	0.6529
SICH at 24–36 h by NINDS	T1	305	5.9% (18/305)	1.00	–	1.00	–
	T2	303	5.6% (17/303)	0.95 (0.48–1.88)	0.8704	1.21 (0.58–2.55)	0.6091
	T3	305	3.9% (12/305)	0.72 (0.34–1.54)	0.4028	0.93 (0.41–2.13)	0.8715
SICH at 24–36 h by ECASS II	T1	305	2.6% (8/305)	1.00	–	1.00	–
	T2	303	2.6% (8/303)	1.04 (0.38–2.83)	0.9374	1.16 (0.42–3.20)	0.7719
	T3	305	3.3% (10/305)	1.58 (0.60–4.14)	0.3543	1.60 (0.58–4.36)	0.3619
SICH at 24–36 h by SITS-MOST	T1	305	0.7% (2/305)	1.00	–	1.00	–
	T2	303	2.0% (6/303)	3.12 (0.62–15.67)	0.1681	3.37 (0.66–17.14)	0.1442
	T3	305	1.3% (4/305)	2.61 (0.46–14.67)	0.2764	2.19 (0.35–13.74)	0.4033

*NINDS, National Institute of Neurological Disorders and Stroke; OR, odds ratio; SITS-MOST, Safe Implementation of Thrombolysis in Stroke-Monitoring Study*.

* *Statistically significant at p < 0.05*.

† *The multivariate logistic regression analysis was adjusted for age, sex, hypertension, and atrial fibrillation*.

### Outcomes by the TyG Index on Continuous Scale

The association between clinical outcomes and the TyG index was explored on a continuous scale (Table 3). The TyG index on a continuous scale showed a significantly increased risk of unfavorable functional outcomes at 90 days in the models of adjustment for age and sex (OR, 1.27, 95% CI, 1.02–1.58; *P* = 0.0361) and of multivariate adjustment (OR, 1.32, 95% CI, 1.01–1.73; *P* = 0.0431). Consistent with the results analyzed by using tertiles, the TyG index was not significantly associated with the outcomes of mortality and SICH within 36 h.

**Table 3:** Functional outcomes and SICH by the TyG index on continuous scale (per unit).

**Models**	***N***	**Events (%)**	**Age, sex- adjusted of TyG index**		**Multivariable- adjusted^†^** **of TyG index**	
**Variable**			**OR (95% CI)**	***p*-value**	**OR (95% CI)**	***p*-value**
Unfavorable functional outcomes at 90 days	813	68.4% (556/813)	1.27 (1.02–1.58)	0.0361^*^	1.32 (1.01–1.73)	0.0431^*^
Mortality at 90 days	813	8.5% (69/813)	1.09 (0.76–1.56)	0.6573	1.16 (0.76–1.76)	0.4959
SICH at 24–36 h by NINDS	914	5.1% (47/914)	0.83 (0.53–1.30)	0.4217	1.00 (0.62–1.62)	0.9947
SICH at 24–36 h by ECASS II	914	2.8% (26/914)	1.24 (0.70–2.19)	0.4698	1.22 (0.67–2.21)	0.5255
SICH at 24–36 h by SITS-MOST	914	1.3% (12/914)	1.43 (0.62–3.29)	0.4065	1.27 (0.51–3.16)	0.6133

*NINDS, National Institute of Neurological Disorders and Stroke; OR, odds ratio; SITS-MOST, Safe Implementation of Thrombolysis in Stroke-Monitoring Study; UFO, unfavorable functional outcome*.

* *Statistically significant at p < 0.05*.

† *The multivariate logistic regression analysis was adjusted for age, sex, hypertension, and atrial fibrillation*.

### Sensitivity Analysis: Stratification by Stroke Severity

**Figure 3:**
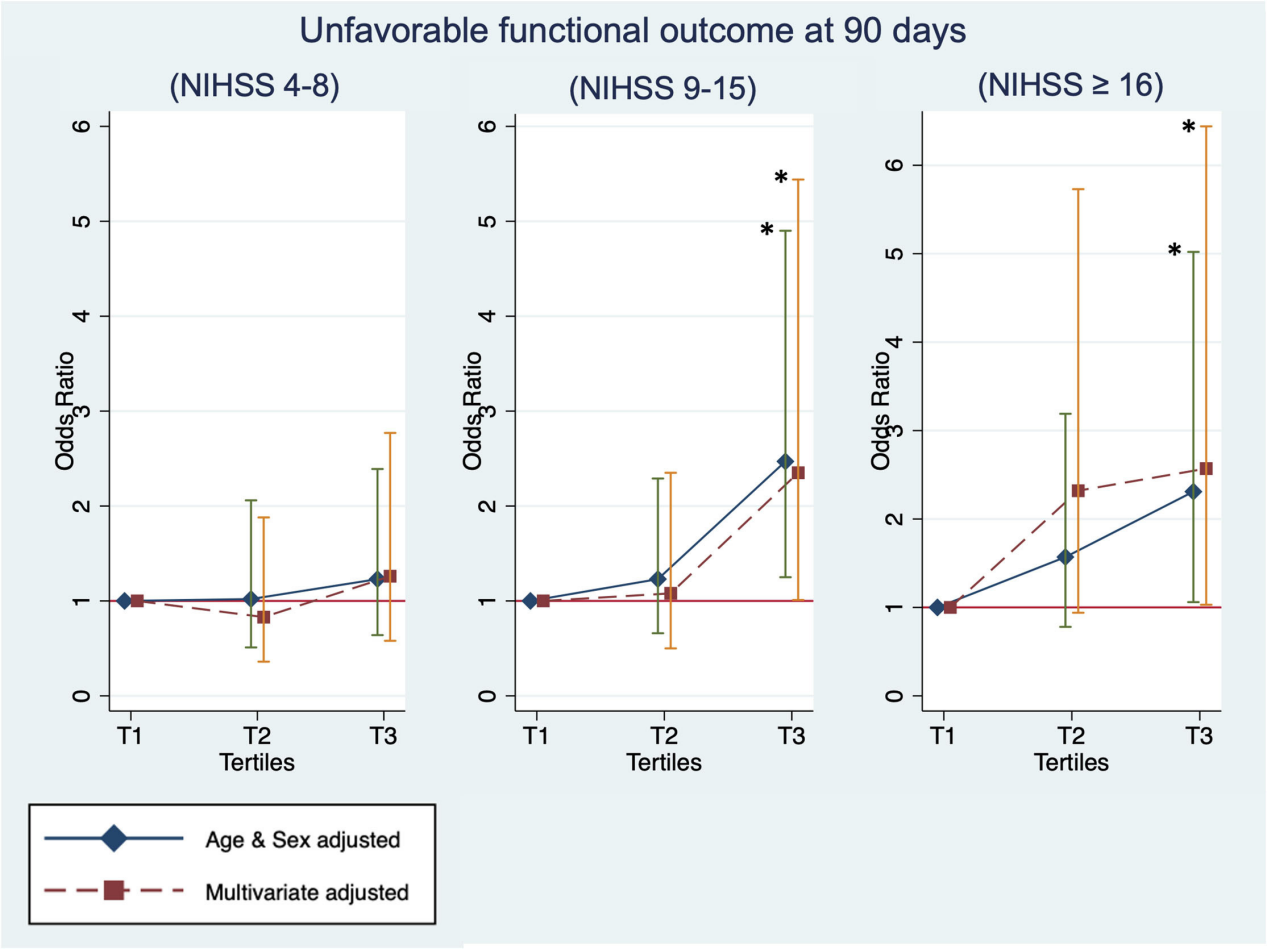
Sensitivity analysis: The unfavorable outcome at 90 days by tertiles of the TyG index and the baseline stroke severity of mild [National Institutes of Health Stroke Scale (NIHSS) of 4–8], moderate (NIHSS of 9–15), and high (NIHSS of ≥16). *Statistical significance (p < 0.05).

The stratification analysis according to stroke severity and tertiles of the TyG index are shown in Table 4. The analysis of unfavorable functional outcomes at 90 days after severe stroke is shown in Figure 3. For patients with mild stroke severity (NIHSS score of 4–8) treated with IVT, no significant association between the TyG index and unfavorable functional outcomes at 90 days was found. In contrast, the T3 category of patients with stroke with moderate severity (NIHSS score of 9–15) showed a 2-fold increased risk of unfavorable functional outcomes at 90 days in both the age and sex-adjusted (OR: 2.47, 95% CI 1.25–4.90, *P* = 0.0096) and multivariate-adjusted regression models (OR, 2.35; 95% CI, 1.01–5.44; *P* = 0.0465). The T3 category of patients with stroke with high stroke severity (NIHSS score of ≥16) showed a two-fold increased risk of unfavorable functional outcomes at 90 days in age and sex-adjusted (OR: 2.31, 95% CI 1.06–5.02, *P* = 0.0355) and multivariate-adjusted regression models (OR: 2.57, 95% CI 1.03–6.44, *P* = 0.0440). Similar to the previous analysis, no significant association was found between the TyG index and outcome measures of mortality and SICH. Lastly, the TyG index on a continuous scale for moderate and severe stroke severity consistently showed an increased risk of unfavorable functional outcomes at 90 days (Table 5).

**Table 4:** Sensitivity analysis: functional outcomes and SICH by tertiles of the TyG index on different stroke severities.

**Models**	**TyG**	***N***	**Events (%)**	**Age, sex- adjusted of TyG index**		**Multivariable-adjusted^†^** **of TyG index**	
**Variable**				**OR (95% CI)**	***p*-value**	**OR (95% CI)**	***p*-value**
Mild severity (NIHSS of 4–8)							
Unfavorable functional outcomes at 90 days	T1	72	41.7% (30/72)	1.00	–	1.00	–
	T2	63	42.9% (27/63)	1.02 (0.51–2.06)	0.9510	0.83 (0.36–1.88)	0.6488
	T3	75	46.7% (35/75)	1.23 (0.64–2.39)	0.5324	1.26 (0.58–2.77)	0.5619
Mortality at 90 days	T1	72	6.9% (5/72)	1.00	–	1.00	–
	T2	63	1.6% (1/63)	0.23 (0.03–2.06)	0.1899	0.78 (0.06–9.72)	0.8463
	T3	75	1.3% (1/75)	0.17 (0.02–1.52)	0.1127	0.71 (0.06–9.26)	0.7962
SICH at 24–36 h by NINDS	T1	81	2.5% (2/81)	1.00	–	1.00	–
	T2	76	2.6% (2/76)	1.11 (0.15–8.16)	0.9196	0.58 (0.05–6.96)	0.6684
	T3	92	2.2% (2/92)	0.89 (0.12–6.56)	0.9100	1.08 (0.13–8.74)	0.9428
SICH at 24–36 h by ECASS II	T1	81	0% (0/81)	1.00	–	1.00	–
	T2	76	0% (0/76)	dispersion	–	dispersion	–
	T3	92	2.2% (2/92)	1.87 (0.24– ∞)^a^	0.3122	1.54 (0.19–∞)	0.3696
SICH at 24–36 h by SITS-MOST	T1	81	0% (0/81)	1.00	–	1.00	–
	T2	76	0% (0/76)	dispersion	–	dispersion	–
	T3	92	1.1% (1/92)	1.00 (0.05–∞) ^a^	0.5000	1.00 (0.05–∞) ^a^	0.5000
Moderate severity (NIHSS of 9–15)							
Unfavorable functional outcomes at 90 days	T1	84	63.1% (53/84)	1.00	–	1.00	–
	T2	105	66.7% (70/105)	1.23 (0.66–2.29)	0.5105	1.08 (0.50–2.35)	0.8483
	T3	97	78.4% (76/97)	2.47 (1.25–4.90)	0.0096^*^	2.35 (1.01–5.44)	0.0465^*^
Mortality at 90 days	T1	84	7.1% (6/84)	1.00	–	1.00	–
	T2	105	2.9% (3/105)	0.37 (0.09–1.55)	0.1711	0.36 (0.08–1.59)	0.1786
	T3	97	3.1% (3/97)	0.45 (0.11–1.92)	0.2815	0.55 (0.12–2.55)	0.4463
SICH at 24–36 h by NINDS	T1	96	7.3% (7/96)	1.00	–	1.00	–
	T2	114	1.8% (2/114)	0.23 (0.05–1.11)	0.0676	0.37 (0.07–2.02)	0.2489
	T3	108	2.8% (3/108)	0.37 (0.09–1.51)	0.1669	0.82 (0.17–3.86)	0.8018
SICH at 24–36 h by ECASS II	T1	96	5.2% (5/96)	1.00	–	1.00	–
	T2	114	0.9% (1/114)	0.16 (0.02–1.43)	0.1021	0.18 (0.02–1.63)	0.1272
	T3	108	2.8% (3/108)	0.61 (0.14–2.72)	0.5166	0.84 (0.17–4.04)	0.8259
SICH at 24–36 h by SITS-MOST	T1	96	1.0% (1/96)	1.00	–	1.00	–
	T2	114	0% (0/114)	0.77 (0.00–14.68)^a^	0.4359	0.50 (0.00–9.50)^a^	0.3333
	T3	108	0.9% (1/108)	0.85 (0.01–83.22)^a^	1.0000	1.50 (0.08–∞)^a^	0.4000
High severity (NIHSS ≥ 16)							
Unfavorable functional outcomes at 90 days	T1	114	78.1% (89/114)	1.00	–	1.00	–
	T2	104	84.6% (88/104)	1.57 (0.78–3.19)	0.2091	2.32 (0.94–5.73)	0.0679
	T3	98	88.8% (87/98)	2.31 (1.06–5.02)	0.0355^*^	2.57 (1.03–6.44)	0.0440^*^
Mortality at 90 days	T1	114	11.4% (13/114)	1.00	–	1.00	–
	T2	104	18.3% (19/104)	1.74 (0.81–3.74)	0.1530	1.65 (0.69–3.92)	0.2594
	T3	98	18.4% (18/98)	1.77 (0.81–3.83)	0.1502	1.71 (0.72–4.08)	0.2272
SICH at 24–36 h by NINDS	T1	127	7.1% (9/127)	1.00	–	1.00	–
	T2	113	11.5% (13/113)	1.77 (0.72–4.36)	0.2136	2.32 (0.88–6.13)	0.0890
	T3	106	6.6% (7/106)	1.03 (0.37–2.91)	0.9545	0.96 (0.61–3.03)	0.9502
SICH at 24–36 h by ECASS II	T1	127	2.4% (3/127)	1.00	–	1.00	–
	T2	113	6.2% (7/113)	3.00 (0.74–12.17)	0.1238	3.38 (0.81–14.20)	0.0959
	T3	106	4.7% (5/106)	2.72 (0.61–12.06)	0.1891	2.09 (0.43–10.24)	0.3652
SICH at 24–36 h by SITS-MOST	T1	127	0.8% (1/127)	1.00	–	1.00	–
	T2	113	5.3% (6/113)	7.51 (0.88–63.93)	0.0650	8.14 (0.94–70.65)	0.0573
	T3	106	1.9% (2/106)	2.89 (0.25–32.94)	0.3928	1.39 (0.08–23.37)	0.8200

*NINDS, National Institute of Neurological Disorders and Stroke; OR, odds ratio; SITS-MOST, Safe Implementation of Thrombolysis in Stroke-Monitoring Study*.

* *Statistically significant at p < 0.05*.

† *The multivariate logistic regression analysis was adjusted for age, sex, hypertension, and atrial fibrillation*.

a *Exact logistic regression model was used*.

**Table 5:** Sensitivity analysis: functional outcomes and SICH by the TyG index on continuous scale (per unit) and different stroke severities.

**Models**	***N***	**Events (%)**	**Age, sex- adjusted of TyG index**		**Multivariable- adjusted^†^** **of TyG index**	
**Variable**			**OR (95% CI)**	***p*-value**	**OR (95% CI)**	***p*-value**
**Mild severity (NIHSS of 4–8)**						
Unfavorable functional outcomes at 90 days	211	44.1% (93/211)	0.99 (0.62–1.59)	0.9709	1.04 (0.64–1.69)	0.8850
Mortality at 90 days	211	3.3% (7/211)	0.99 (0.23–4.30)	0.9912	1.35 (0.32–5.62)	0.6807
SICH at 24–36 h by NINDS	250	2.4% (6/250)	1.06 (0.32–3.55)	0.9290	1.23 (0.34–4.39)	0.7547
SICH at 24–36 h by ECASS II	250	0.8% (2/250)	2.54 (0.48–13.58)	0.2763	2.52 (0.42–15.19)	0.3144
SICH at 24–36 h by SITS-MOST	250	0.4% (1/250)	3.80 (0.19–76.59)	0.3838	1.00 (0.10–∞)^a^	0.5000
**Moderate severity (NIHSS of 9–15)**						
Unfavorable functional outcomes at 90 days	286	69.6% (199/286)	1.38 (0.85–2.25)	0.1983	1.43 (0.87–2.36)	0.1575
Mortality at 90 days	286	4.2% (12/286)	0.46 (0.17–1.25)	0.1285	0.50 (0.18–1.40)	0.1877
SICH at 24–36 h by NINDS	318	3.8% (12/318)	0.79 (0.28–2.19)	0.4217	0.96 (0.31–2.99)	0.9491
SICH at 24–36 h by ECASS II	318	2.8% (9/318)	0.79 (0.27–2.32)	0.6705	0.95 (0.28–3.18)	0.9280
SICH at 24–36 h by SITS-MOST	318	0.6% (2/318)	2.06 (0.24–17.59)	0.5091	8.99 (0.22–376.14)	0.2491
**High severity (NIHSS of** **≥16)**						
Unfavorable functional outcomes at 90 days	316	83.5% (264/316)	1.77 (1.02–3.07)	0.0431^*^	1.88 (1.07–3.33)	0.0295^*^
Mortality at 90 days	316	15.8% (50/316)	1.42 (0.88–2.29)	0.1481	1.40 (0.86–2.29)	0.1713
SICH at 24–36 h by NINDS	346	8.4% (29/346)	0.99 (0.56–1.77)	0.9736	0.97 (0.54–1.74)	0.9041
SICH at 24–36 h by ECASS II	346	4.3% (15/346)	1.22 (0.56–2.65)	0.6120	1.19 (0.55–2.54)	0.6593
SICH at 24–36 h by SITS-MOST	346	2.6% (9/346)	0.95 (0.34–2.71)	0.9302	0.94 (0.33–2.62)	0.9006

*NINDS, National Institute of Neurological Disorders and Stroke; OR, odds ratio; SITS-MOST, Safe Implementation of Thrombolysis in Stroke-Monitoring Study*.

* *Statistically significant at p < 0.05*.

† *The multivariate logistic regression analysis was adjusted for age, sex, hypertension, and atrial fibrillation*.

a *Exact logistic regression model was used*.

## Discussion

Our results showed that the TyG index was associated with unfavorable functional outcomes at 90 days in patients with acute ischemic stroke treated with IVT. This association was more robust in patients with stroke presenting with moderate to high baseline severity. In addition, there was no significant association between the TyG index and outcomes of mortality or SICH within 36 h.

Our analysis employing the TyG index corroborated the results of previous studies. In an earlier investigation, higher resistance to IVT therapy and poor clot dissolution were observed in patients with stroke with metabolic syndrome under transcranial Doppler examination (30, 31). An earlier study showed that HOMA-IR in the upper tertile was associated with eight-fold increased risk of unfavorable functional outcome when compared with the lower tertile (29). In addition, a recent large-scale study that enrolled nondiabetic Chinese patients with stroke found that higher HOMA-IR was linked to poor stroke recovery and recurrence (19). These studies suggested that patients with stroke presenting with higher IR should be poor responders to IVT and more susceptible to unfavorable recovery. This study uncovers important implications that the TyG index, such as HOMA-IR, is an effective biomarker for selecting patients who benefit from aggressive strategies for vascular reperfusion. Unlike HOMA-IR, the TyG index is more practical for use in clinical conditions due to the lack of insulin measurement.

Recent studies have suggested that the TyG index is highly associated with carotid atherosclerosis (15), major adverse cardiovascular events in patients with non-ST-segment elevation acute coronary syndrome (ACS), and major adverse cardiovascular and cerebrovascular events in patients with ST-segment elevation ACS (16, 17). The above studies support that the TyG index is a marker for acute adverse effects in cardiovascular and cerebrovascular diseases. In terms of physiology, high values of the TyG index or IR should oppose IVT for ischemic stroke by several mechanisms. First, several studies reported that higher IR was associated with elevated levels of thrombin activatable fibrinolysis inhibitor and plasminogen activator inhibitor 1 (PAI-I) (32, 33), which attenuated fibrinolysis by IVT (33). Second, IR augments the density of blood clots and impairs the effect of IVT (34).

Our results showed no significant association between the TyG index and SICH outcomes and are compatible with our previous analysis of SICH outcomes on TG levels in the TTT-AIS study (35). This findings were consistent with an earlier study that used HOMA-IR for patients with stroke treated with IVT (29). Based on previous studies of TG, which did not show robust association with intracranial hemorrhage in patients with stroke treated without thrombolysis (36–40), we deduced that the TyG index was not a reliable biomarker for hemorrhagic transformation. Additionally, the TyG index showed no significant association with the outcome of mortality. The most reasonable explanation was that two-thirds of patients with stroke presented with moderate and high severity and these patients were more susceptible to SICH with IVT when compared to those with low severity or with the low NIHSS score (41–44). In a separate analysis, the mortality in our cases was also strongly attributable to SICH (Supplementary Table S1).

Our cutoff values for tertiles of the TyG index at 8.48 and 9.04 should be used as a standard. A recent study investigating patients with type 2 diabetes mellitus found that the TyG index >9.5 significantly increased macrovascular complications, including cerebrovascular diseases and albuminuria (45). Another study that enrolled patients with minor cases (the median NIHSS score, 4; quartile deviation, 2.5) reported that the TyG index of >9.2 increased neurological worsening (21). A cross-sectional study exploring silent brain infarcts reported increased multiple silent infarcts in the group with the median TyG index >8.5 (46). To the best of our knowledge, patients with acute ischemic stroke with the TyG index >9 should be cautious of worsening clinical neurological function.

We confirmed that the TyG index was robust in predicting unfavorable functional outcomes at 90 days for moderate and severe stroke severity. This was also the first study to explore the TyG index for different stroke severities. In terms of physiology, we proposed that the harder and longer segments of clots in patients with stroke with high severity would be aggravated by attenuated fibrinolysis due to increased IR. In addition, the distinctive strengths of this study are as follows: (1) a longitudinal cohort study design with a large sample size of patients with stroke treated with IVT, (2) determination of the extent to which a higher level of the TyG index contributes to unfavorable functional outcomes at 90 days, and (3) estimation of outcomes using the TyG index on tertile and continuous scales, with robust results in sensitivity analysis.

Overall, our results are consistent to earlier studies investigating the effect of IR in patients with acute ischemic stroke. Due to the need of insulin measurement, use of HOMA-IR in the real-world practice is limited. For patients with stroke, the lipid profile and blood glucose are routine laboratory tests. Therefore, physicians can easily monitor the effect of IR in patients with stroke with the TyG index. However, further study is warranted to evaluate whether controlling the TyG index <9.0 would improve the functional outcomes and accelerate neurological recovery. More evidence is needed to generalize the results for clinical practice in Asians and other ethnic population.

Stress hyperglycemia and IR are reported as the adaptive response that increase the chance of the patient to survive (47). Chronic hyperglycemia is known harmful with numerous complications (48, 49). Recently, acute hyperglycemia has been considered protective, since patients could have greater cellular resistance to ischemic and hypoxic injury (47, 50). In terms of pathophysiology, stress hyperglycemia is caused predominantly by excessive gluconeogenesis, glycogenolysis, and IR. While epinephrine and norepinephrine augment hepatic gluconeogenesis and glycogenolysis, inflammatory cytokines, including tumor necrosis factor-α (TNF-α), interleukin-6 (IL-6), and C-reactive protein, induce IR (51, 52). In addition, glucose is the primary energy source of the brain (53). Therefore, we consider that stress hyperglycemia should not be excluded, since it represents the physiological response to improve survival in patients with stroke.

This study had some limitations. First, the TTT-AIS registry (22, 23) has no clinical data on insulin levels. Further, HOMA-IR was not used in this study. Second, the TTT-AIS registry was initiated in 2004 at a time when thrombectomy had not been introduced in Taiwan. Third, although we have conducted the subgroup analysis in diabetic and nondiabetic patients according to medical history, no significant association between functional outcomes and the TyG index was found (Supplementary Table S2). Two factors should explain this: (1) history of diabetes mellitus reported by patients was imprecise and underdiagnosed and (2) the smaller sample size in subgroup analysis. However, a recently published study (54) showed that the TyG index was associated with early neurological deterioration (an increase of the NIHSS ≥2 or the NIHSS ≥1 in the motor dysfunction within 72 h) in patients with untreated diabetes (adjusted OR: 3.94, 95% CI, 1.47–10.53, *P* = 0.006). Accordingly, the TyG index should be applicable in predicting functional outcome in patients with stroke with diabetes mellitus. Fourth, hemoglobin A1c was not measured in this study. The prevalence of diabetes mellitus was possibly underdiagnosed in this study.

## Conclusion

In conclusion, this study supports a strong association between higher levels of the TyG index and increased unfavorable functional outcomes at 90 days in patients with acute ischemic stroke treated with IVT. This association was robust in patients with moderate (NIHSS of 9–15) and high stroke severity (NIHSS ≥ 16). While HOMA-IR is not readily available, the TyG index would be a surrogate marker of IR. This study determines a cutoff value of TyG index >9.0 that is useful for predicting unfavorable functional outcomes in patients with acute ischemic stroke treated with IVT in Asians, but further study is needed to validate this cutoff value in other ethnic population.

## Data Availability Statement

The data analyzed in this study is subject to the following licenses/restrictions: The datasets presented in this article are not readily available because the Institutional Review Board of Kaohsiung Medical University has restricted their distribution. Requests to access these datasets should be directed to A-Ching Chao, achch@cc.kmu.edu.tw.

## Ethics Statement

The studies involving human participants were reviewed and approved by Kaohsiung Medical University Hospital (IRB: KMUH-IRB-20140305). The patients/participants provided their written informed consent to participate in this study.

## Author Contributions

S-FL wrote the first draft of the article. All authors contributed to the conception and design of the study, acquisition, analysis, and interpretation of data.

## Funding

This study was supported by grants from the Ministry of Science and Technology (Reference Number: MOST 108-2314-B-037-038-MY3) and the Kaohsiung Medical University Hospital (Reference Number: KMUH110-0R65).

## Conflict of Interest

The authors declare that the research was conducted in the absence of any commercial or financial relationships that could be construed as a potential conflict of interest.

## Publisher's Note

All claims expressed in this article are solely those of the authors and do not necessarily represent those of their affiliated organizations, or those of the publisher, the editors and the reviewers. Any product that may be evaluated in this article, or claim that may be made by its manufacturer, is not guaranteed or endorsed by the publisher.
