# Supplementary material for: Triglyceride-Glucose Index and Intravenous Thrombolysis Outcomes for Acute Ischemic Stroke: A Multicenter Prospective–Cohort Study
Source: Front Neurol. 2022 Feb 14;13:737441. doi: 10.3389/fneur.2022.737441 (PMC8890321; doi:10.3389/fneur.2022.737441)
Supplement: Supplementary file 1 [file Data_Sheet_1.PDF]

Table S1. Association between Mortality and Symptomatic Intracranial Hemorrhage.

Models	Mortality (unadjusted)		Mortality (Age-, sex-adjusted)	
Variable	OR (95% CI)	*p* value	OR (95% CI)	*p* value
SICH by NINDS	5.73 (2.82–11.63)	< 0.0001	5.38 (2.62–11.03)	< 0.0001
SICH by ECASS II	8.44 (3.47–20.53)	< 0.0001	8.28 (3.32–20.64)	< 0.0001
SICH by SITS-MOST	17.62 (4.85–64.07)	< 0.0001	16.28 (4.39–60.36)	< 0.0001

CI, confidence interval; NINDS, National Institute of Neurological Disorders and Stroke; OR, odds ratio; SITS-MOST, Safe Implementation of Thrombolysis in Stroke-Monitoring Study; *Statistically significant at $p<0.05$.

Table S2. Subgroup Analysis between Diabetic and Non-diabetic Patients According to Medical History

Models			Age, sex-adjusted of TyG index		Multivariable-adjusted† of TyG index	
Variable	*N*	Events (%)	OR (95% CI)	*p* value	OR (95% CI)	*p* value
Diabetic patients (*N* = 316)						
Unfavorable functional outcomes at 90 days	280	74.7% (209/280)	1.17 (0.79–1.74)	0.4251	1.21 (0.76–2.12)	0.3592
Mortality at 90 days	280	11.1% (31/280)	1.10 (0.63–1.91)	0.7440	1.40 (0.72–2.73)	0.3183
SICH at 24–36 h by NINDS	316	5.7% (18/316)	1.02 (0.49–2.12)	0.9657	0.97 (0.45–2.11)	0.9461
SICH at 24–36 h by ECASS II	316	3.8% (12/316)	1.25 (0.51–2.93)	0.6449	1.12 (0.44–2.84)	0.8179
SICH at 24–36 h by SITS-MOST	316	2.5% (8/316)	1.56 (0.52–4.65)	0.4256	1.36 (0.41–4.53)	0.6168
Non-diabetic patients (*N* = 598)						
Unfavorable functional outcomes at 90 days	533	65.1% (347/533)	1.12 (0.82–1.53)	0.4791	1.15 (0.81–1.64)	0.4297
Mortality at 90 days	533	7.1% (38/533)	0.77 (0.43–1.39)	0.3887	0.80 (0.42–1.51)	0.4883
SICH at 24–36 h by NINDS	598	4.8% (29/598)	0.66 (0.34–1.28)	0.2205	0.87 (0.43–1.75)	0.6859
SICH at 24–36 h by ECASS II	598	2.3% (14/598)	1.06 (0.43–2.61)	0.9017	1.12 (0.45–2.76)	0.8125
SICH at 24–36 h by SITS-MOST	598	1.0% (4/598)	0.38 (0.06–2.57)	0.3235	0.39 (0.05–2.92)	0.3572

CI, confidence interval; NINDS, National Institute of Neurological Disorders and Stroke; OR, odds ratio; SITS-MOST, Safe Implementation of Thrombolysis in Stroke-Monitoring Study; TyG, Triglyceride-glucose index; UFO, unfavorable functional outcome *Statistically significant at $p<0.05$.
†Multivariable regression was adjusted for age, sex, hypertension, and atrial fibrillation.
